# WizePairZ: a novel algorithm to identify, encode, and exploit matched molecular pairs with unspecified cores in medicinal chemistry

**DOI:** 10.1186/1758-2946-3-S1-O9

**Published:** 2011-04-19

**Authors:** Dan J Warner, Stephen A St-Gallay, Edward J Griffen

**Affiliations:** 1 AstraZeneca, Loughborough, UK; 2 AstraZeneca, Alderly Park, UK

## 

An algorithm to automatically identify and extract matched molecular pairs from a collection of compounds has been developed, allowing the learning associated with each molecular transformation to be readily exploited in drug discovery projects. Here, we present the application to an example data set of 11 histone deacetylase inhibitors. The matched pairs were identified, and corresponding differences in activity and lipophilicity were recorded. These property differences were associated with the chemical transformations encoded in the SMIRKS reaction notation. The transformations identified a subseries with the optimal balance of these two parameters. Enumeration of a virtual library of compounds using the extracted transformations identified two additional compounds initially excluded from the analysis with an accurate estimation of their biological activity. We describe how the WizePairZ system can be used to archive and apply medicinal chemistry knowledge from one drug discovery project to another as well as identify common bioisosteres.

